# A Common Left Occipito-Temporal Dysfunction in Developmental Dyslexia and Acquired Letter-By-Letter Reading?

**DOI:** 10.1371/journal.pone.0012073

**Published:** 2010-08-11

**Authors:** Fabio Richlan, Denise Sturm, Matthias Schurz, Martin Kronbichler, Gunther Ladurner, Heinz Wimmer

**Affiliations:** 1 Department of Psychology and Center for Neurocognitive Research, University of Salzburg, Salzburg, Austria; 2 Department of Neurology, Christian Doppler Clinic, Paracelsus Private Medical University, Salzburg, Austria; University of Leuven, Belgium

## Abstract

**Background:**

We used fMRI to examine functional brain abnormalities of German-speaking dyslexics who suffer from slow effortful reading but not from a reading accuracy problem. Similar to acquired cases of letter-by-letter reading, the developmental cases exhibited an abnormal strong effect of length (i.e., number of letters) on response time for words and pseudowords.

**Results:**

Corresponding to lesions of left occipito-temporal (OT) regions in acquired cases, we found a dysfunction of this region in our developmental cases who failed to exhibit responsiveness of left OT regions to the length of words and pseudowords. This abnormality in the left OT cortex was accompanied by absent responsiveness to increased sublexical reading demands in phonological inferior frontal gyrus (IFG) regions. Interestingly, there was no abnormality in the left superior temporal cortex which—corresponding to the onological deficit explanation—is considered to be the prime locus of the reading difficulties of developmental dyslexia cases.

**Conclusions:**

The present functional imaging results suggest that developmental dyslexia similar to acquired letter-by-letter reading is due to a primary dysfunction of left OT regions.

## Introduction

Recent studies on the manifestation of developmental reading problems established an interesting behavioral similarity to a form of acquired dyslexia referred to as letter-by-letter (LBL) reading. As suggested by the term letter-by-letter reading, the critical manifestation is an abnormal effect of word length, that is, number of letters, on reading time. To illustrate, Cohen et al. [1], reported linear increases of reading time with increases in word length in acquired LBL readers, ranging up to 400 ms per each additional letter in words from 3 to 9 letters. Similar, although less dramatic, length related reading latency increases were reported for English, German, and Italian dyslexic children and adolescents [2]–[4]. An abnormal word-length effect of developmental dyslexia cases was not only observed for latencies of reading aloud responses but also for visual inspection time in eye movement studies [5], [6]. In terms of the prominent cognitive dual-route model of visual word processing (e.g., [7]), both the loss of efficient (i.e., length-independent) visual word processing in acquired LBL reading and the difficulty to attain efficient word processing in developmental dyslexia may be traced to a dysfunction of the lexical reading route. The critical component of the lexical route is the orthographic word lexicon which contains representations of the letter sequence of frequently read words. Such orthographic word entries allow fast visual whole-word recognition, that is, parallel assimilation of letter strings and direct access to word phonology and meaning. Frequent absence of such orthographic word entries results in reliance on the slow sublexical reading route. This route achieves access to word phonology by serial sublexical orthographic-phonological recoding which obviously gives rise to the abnormal length effect on reading time for words. However, developmental dyslexia cases suffer not only from an abnormal length effect for words, but they also exhibit inefficient sublexical processing of the unfamiliar letter strings of pseudowords (e.g., [8]), which may be attributed to frequent absence of larger sublexical multi-letter recognition units and adherence to serial grapheme-phoneme coding instead. Furthermore, even when dyslexic readers rely on lexical route processing of words, they still were found to exhibit a reading speed deficit [6], [9]–[11].

In the field of acquired dyslexia, there is growing consensus that the loss of efficient word processing and the emergence of LBL reading is caused by lesions affecting the left ventral occipito-temporal (OT) cortex, specifically the Visual Word Form Area (VWFA), or connections to or from the VWFA [1], [12]–[16]. Similarly, one may hypothesize that the difficulty with fast fluent reading of developmental dyslexia cases may be caused by a congenital dysfunction of the OT cortex. However, this hypothesis is quite different from the dominant explanatory framework in the field of developmental dyslexia (e.g., [17]). Here, the main dyslexic difficulty is seen in the acquisition of the sublexical route, that is, in self-reliant phonological word decoding. This difficulty is seen as arising from a verbal-phonological deficit which affects the identification of phonemes in spoken words which, in turn, affects the mapping of graphemes onto phonemes which, in turn, affects the acquisition of self-reliant phonological word decoding which, in turn, affects orthographic learning. In correspondence with the phonological deficit explanation, reviews of imaging studies summarize the findings as speaking for a primary dysfunction of posterior language areas (i.e., posterior superior temporal gyrus/sulcus and adjacent parietal regions) and consider underactivation of left OT regions as secondary to the primary dysfunction of left temporo-parietal (TP) region [18]–[22].

Evidence for the phonological deficit explanation and specifically for a profound dyslexic difficulty with the acquisition of self-reliant word decoding is largely based on English language which is an outlier with respect to grapheme-phoneme regularity [23]. In more typical alphabetic orthographies with transparent grapheme-phoneme correspondences, reading accuracy for short words and pseudowords approaches ceiling after a couple of months of instruction [24], [25]. Even for dyslexic children, the mentioned reading fluency impairment typically occurs in the context of high reading accuracy (e.g., Dutch: [26], Finnish: [27], Greek: [28], Hebrew: [29], Italian: [30], Norwegian: [31], Spanish: [32]). Direct German-English comparisons with similar words and pseudowords confirmed the difference in reading accuracy [33], [34]. To illustrate, for low frequency words, Landerl et al. [33] found that accuracy was about 93% for German dyslexic children compared to only about 50% for their English peers. The ease of accurate phonological word reading in regular orthographies is of theoretical importance as it raises doubts that poor orthographic learning (i.e., reduced storage of letter strings for words or larger segments) is secondary to difficulty with accurate phonological reading. This then raises further doubts whether observed dysfunctions of the left OT regions in dyslexic readers are secondary to a primary dysfunction of left posterior language regions. The alternative possibility is that the fluency problem of dyslexic readers in regular orthographies – similar to acquired cases of LBL reading – may be caused by a primary dysfunction of left OT regions engaged by highly efficient lexical and sublexical route processes. There is already some support for this latter hypothesis from functional imaging studies with German-speaking dyslexic children and adults who suffer from the characteristic reading speed problem [35]–[38].

The present fMRI study extended this line of research by focusing specifically on dyslexic abnormalities in the brain response to increased length (number of letters) of words and pseudowords. As mentioned above, dyslexic readers similar to LBL readers exhibited abnormal increases of reading time with increasing word length. For studying abnormal brain responses, we extended a recent imaging study with nonimpaired readers from our lab [39] by adding a dyslexic sample. This study manipulated item length with short items (words and pseudowords) consisting of 3–5 letters and long ones of 6–10 letters. With respect to the mentioned reviews of imaging studies, several findings of Schurz et al. [39] are important. Firstly, the processing of both words and pseudowords led to marked activation in left OT and left inferior frontal gyrus (IFG) regions, but not in left temporal regions. Secondly, the activation pattern in both the OT and the IFG regions corresponded to expectations from lexical processing of words and sublexical processing of pseudowords as there was no effect of length on the brain response to words, but a substantial effect in the response to pseudowords. Of main interest with respect to a primary or secondary dysfunction of left OT regions is the activation pattern shown by the present dyslexic sample. Let us first consider expectations from an only secondary dysfunction, that is, less engagement by lexical whole-word recognition and sublexical multi-letter recognition of otherwise fully functional left OT regions. From this hypothesis one would expect left OT activation to correspond to the behavioral response pattern shown by our dyslexic sample. Compared to the nonimpaired sample, our dyslexic readers exhibited generally prolonged response latencies and a stronger length effect on response latencies for both words and pseudowords. When fully functional left OT regions of dyslexic readers are engaged by slow serial letter string processing resulting in the mentioned latency pattern, one would expect increased activation and a stronger length effect on activation. This pattern is not expected from a primary dysfunction of left OT regions. From this hypothesis one would expect – compared to controls – generally reduced activation and, specifically, absence of a length effect on activation.

## Results

### Behavioral results

For interpretation of brain activation findings, dyslexic abnormalities in the effect of item length on response latencies for words and pseudowords are important. The words and pseudowords were presented together with pseudohomophones in a phonological lexical decision task (i.e., “Does xxx sound like a real word?”). This makes unlikely that the NO response to pseudowords could be based on orthographic familiarity checks because such a strategy would have resulted in NO responses to both pseudohomophones and pseudowords. This was not the case as percentages of YES responses to pseudohomophones were 84% and 76% for nonimpaired and dyslexic readers, respectively. In the preceding Schurz et al. [39] study, pseudohomophones were excluded from analysis because short pseudohomophones led to a higher number of erroneous NO responses than long ones and because there was no length effect on response times. The reason for the specific difficulty of short pseudohomophones may have been overly accurate pronunciations. For example, such a pronunciation in the case of “Prot” (instead of “Brot” – bread) may have led to a NO response or to repeated processing before arriving at a YES response. In the case of long pseudohomophones such as “Broduktion” (instead of “Produktion” – production) overly accurate pronunciation may have been less distracting.

The response latencies in Table 1 show that dyslexic readers exhibited generally prolonged response latencies and were more negatively affected by item length (short vs. long) as evident from a reliable main effect of group, *F*(1, 31) = 5.41, *p*<.05, and a reliable length by group interaction, *F*(1, 31) = 26.74, *p*<.001. The lexicality (words vs. pseudowords) by group interaction was of borderline significance, *F*(1, 31) = 3.25, *p* = .08. The three-way interaction was not reliable, *F*(1, 31) = 1.97, *p* = .17. Of specific interest is that the latency increase exhibited by dyslexic readers from short to long words of about 170 ms was more than tripled compared to the small increase of about 50 ms exhibited by the nonimpaired sample. The mean percentages of correct responses in Table 1 show that dyslexic readers had little difficulty with words (over 90% correct YES responses) but exhibited some difficulty to reject pseudowords. The difference was reliable for short pseudowords, *t*(31) = 2.39, *p*<.05, and increased for long pseudowords as evident from a length by group interaction, *F*(1, 31) = 4.24, *p*<.05.

**Table 1:** Means and standard deviations of in-scanner performance.

		Nonimpaired readers	Dyslexic readers
		(*n* = 18)	(*n* = 15)
Speed (ms)			
Words	short	848 (253)	971 (267)
	long	894 (270)	1139 (336)
	length effect	46 (41)	168 (88)
Pseudowords	short	1080 (283)	1322 (348)
	long	1219 (283)	1537 (341)
	length effect	139 (66)	215 (88)
Accuracy (% correct)			
Words	short	94.89 (6.91)	93.53 (6.82)
	long	96.22 (5.64)	90.47 (8.58)
	length effect	−1.33 (3.69)	3.07 (6.40)
Pseudowords	short	90.72 (9.20)	80.80 (14.49)
	long	87.94 (11.17)	73.20 (15.90)
	length effect	2.78 (7.09)	7.60 (6.20)

### Imaging results

In the first section we report whole-brain analyses with a focus on dyslexic activation abnormalities. In the following sections, regions of interest (ROIs) examined group differences (a) along the left ventral visual pathway including critical OT regions, (b) in left TP regions which were hypothesized to be dysfunctional in dyslexic readers, and (c) in left frontal language areas which were identified with dyslexic abnormalities in the whole-brain analyses.

#### Whole-brain analyses

**Figure 1:**
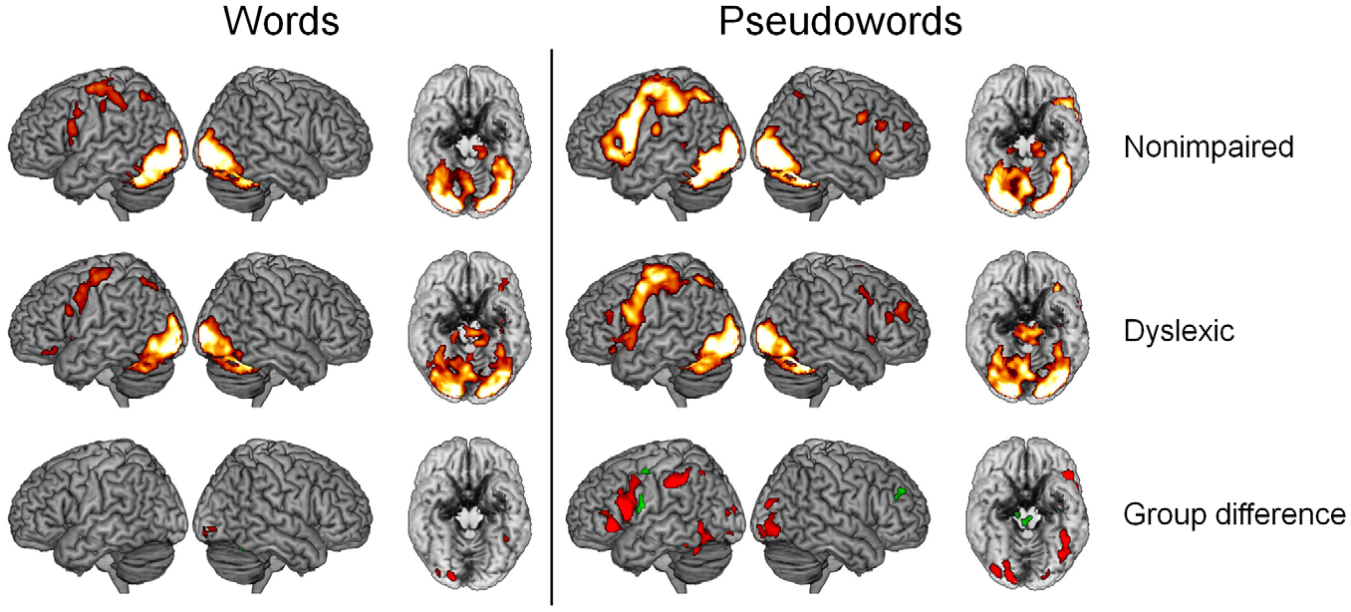
Whole brain activation. Brain renders of the within- and between group whole-brain results showing dyslexic under- (red) and overactivation (green) in response to words vs. fixation and pseudowords vs. fixation, respectively.

The results of these analyses are illustrated in Figure 1 and reported in detail in Table 2. Figure 1 shows that for words (short and long items combined vs. baseline) both groups exhibited highest activation levels in the occipital cortex with activations extending into occipito-parietal and occipito-temporal regions. There was activation in left frontal and parietal regions and in bilateral subcortical and cerebellar regions as well. A main result of the group comparisons is that dyslexic readers exhibited underactivation (red) of a small OT cluster with a maximum difference at (MNI-coordinates) x = −42, y = −46, z = −16. The only other region with underactivation was centered in the right inferior occipital gyrus. These underactivations stood in contrast to overactivation (green) in the left supplementary motor area (SMA), the left lingual gyrus, and the right cerebellum. The largest regions with overactivation were identified in subcortical structures (bilateral thalamus, bilateral caudate, left putamen, pallidum, and amygdala).

**Table 2:** Results of the whole-brain group comparisons.

	MNI-coordinates			Volume	
Region	x	y	z	(voxels)	*Z*
Words > Baseline					
Nonimpaired > Dyslexic					
R inferior occipital	22	−90	−6	100	4.84
	36	−86	−6	20	2.91
L VWFA	−42	−46	−16	23	3.44
Dyslexic > Nonimpaired					
L lingual	−16	−80	8	22	3.99
L SMA	−2	4	54	114	3.53
Subcortical structures					
L thalamus	−14	−20	12	1073	4.63
L putamen	−16	0	10	748	4.48
R caudate	18	16	6	139	4.13
R cerebellum	30	−56	−30	60	3.76
Pseudowords > Baseline					
Nonimpaired > Dyslexic					
Posterior brain regions					
R inferior occipital	22	−90	−4	334	5.68
	36	−84	−6	227	3.92
L lingual	−18	−92	−8	32	3.06
R middle occipital	24	−86	18	113	4.14
L cuneus	−16	−92	16	97	4.03
L angular	−34	−62	50	26	3.55
L supramarginal	−44	−44	40	274	4.78
L VWFA	−44	−48	−18	487	4.82
L middle temporal	−46	−46	4	100	4.51
Frontal brain regions					
L IFG opercular	−52	14	14	904	5.25
R IFG opercular	36	4	28	52	4.00
L IFG triangular	−48	28	−4	277	5.10
R insula	34	22	12	29	2.92
L medial frontal	−2	24	44	22	3.38
Anterior cingulum	10	30	26	49	3.46
Dyslexic > Nonimpaired					
L precuneus	−16	−66	40	47	3.61
Frontal brain regions					
R SMA	16	2	56	370	4.57
L superior frontal	−20	2	58	36	4.25
L precentral	−58	−2	16	58	3.64
	−48	−4	52	28	3.28
R middle frontal	32	44	34	37	3.34
Subcortical structures					
R putamen	18	2	10	144	4.46
L caudate	−18	14	12	89	4.42
R caudate	16	18	6	21	3.38
L thalamus	−12	−6	−6	52	4.14
	−8	−22	16	217	3.64
L hippocampus	−38	−12	−24	21	3.59
L amygdala	−28	−8	−10	20	3.33
L pallidum	−20	−2	8	24	2.97
L cerebellum	−28	−50	−32	156	4.11
R cerebellum	10	−58	−22	37	3.56
	32	−54	−32	66	3.47
R brainstem	14	−18	−8	23	3.04

Pseudowords activated largely similar regions as words, but activation – specifically in the left frontal cortex – was much higher and more extended. Compared to words, a higher number and more extended regions were identified with dyslexic underactivation. Specifically, the right occipital underactivation was more extended, and the same was the case for the underactivation in the left OT region with an additional maximum in the posterior middle temporal gyrus (x = −46, y = −46, z = 4). Further large regions with underactivation were identified in left inferior parietal and in left inferior frontal regions. Regions with overactivation were found in the left precentral gyrus and in the right middle frontal gyrus. Large regions with overactivation were identified in bilateral aspects of the SMA, in subcortical regions (putamen, thalamus), and in cerebellar regions.

#### Left occipito-temporal (OT) regions

**Figure 2:**
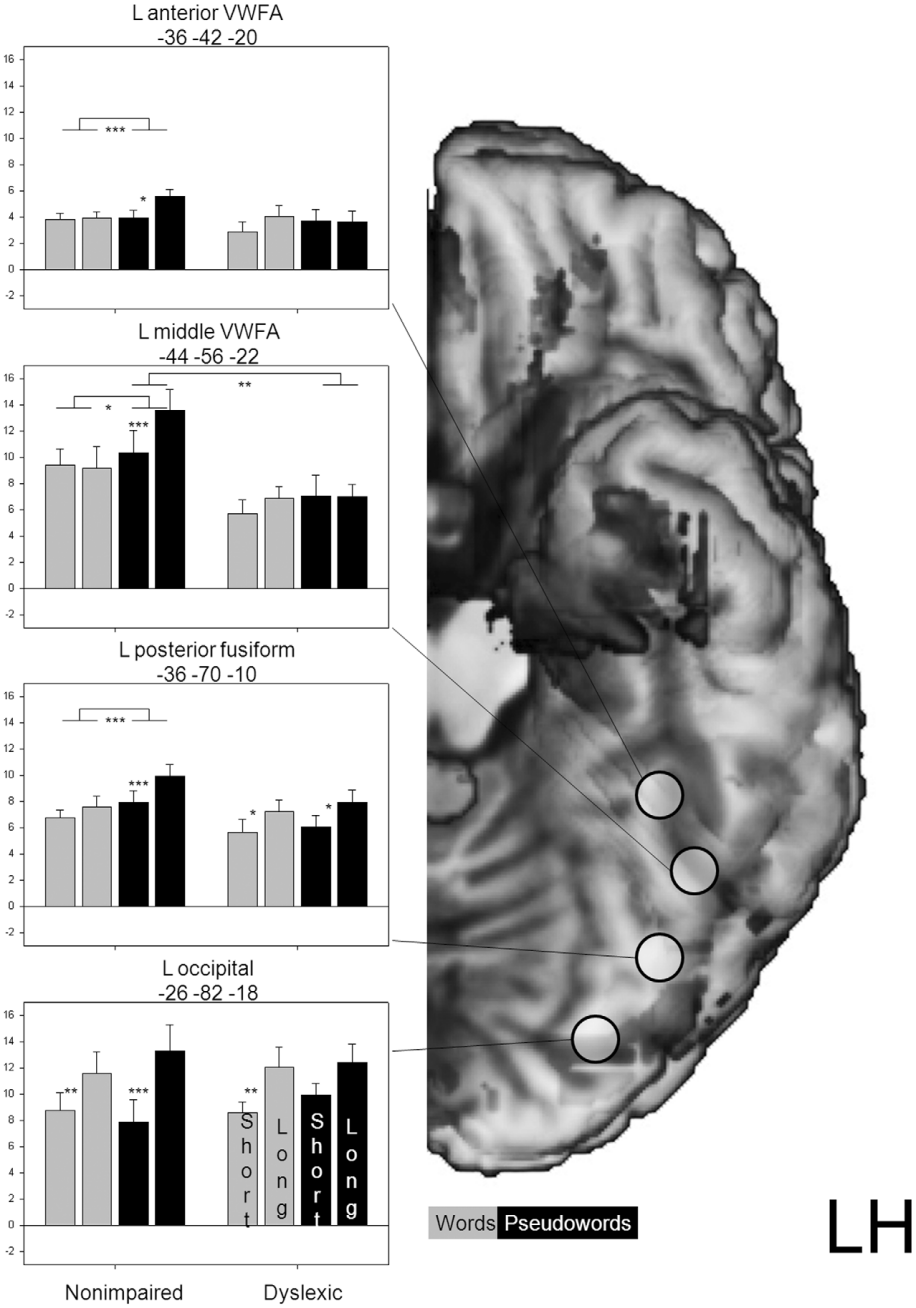
Activation in the left ventral visual pathway. Means and standard errors of extracted signal change estimates (in arbitrary units) and approximate locations of ROIs in the left ventral visual pathway. Statistically reliable effects are indicated by asterisks. * *p*<.05, ** *p*<.01, *** *p*<.001.

Given the focus on dyslexic abnormalities in the left OT cortex, we selected two ROIs in the OT sulcus corresponding to a middle (y = −56) and an anterior segment of the VWFA (y = −42) of Cohen et al. [40]. Furthermore, we included a ROI in the posterior fusiform gyrus (y = −70). Figure 2 shows the location of the ROIs (5-mm-radius spheres) and the corresponding coordinates. These regions were identified by the preceding Schurz et al. [39] study as exhibiting maxima of reading related activation (i.e., comparing activation to all item types against baseline) in nonimpaired readers. These ROIs were used to extract percent signal change estimates (in arbitrary units) for each of the four item types in each participant. The main finding is that in the two anterior ROIs, dyslexic readers exhibited a strikingly different activation pattern compared to nonimpaired readers as they failed to exhibit the marked length effect for pseudowords shown by the nonimpaired readers. In the two posterior ROIs, the dyslexic readers, similar to the controls, exhibited length effects for both words and pseudowords, *p*s<.05. Because of rather high activation in response to short pseudowords, the length effect for pseudowords in the occipital ROI was of borderline reliability, *p* = .07.

A possible concern is that the absence of a length effect for dyslexic readers in the two left anterior OT ROIs (x = −36, y = −42, z = −20, and x = −44, y = −56, z = −22) is due to the fact that these ROIs were based on the nonimpaired sample only. We redid the ROI analysis based on left OT maxima of reading related activation in the combined sample of nonimpaired and dyslexic readers. The results were close to identical with the original analysis, which is not surprising given that these ROIs at x = −36, y = −42, z = −22, and x = −44, y = −56, z = −24 were very close to those from the nonimpaired readers.

#### Left temporo-parietal (TP) regions

**Figure 3:**
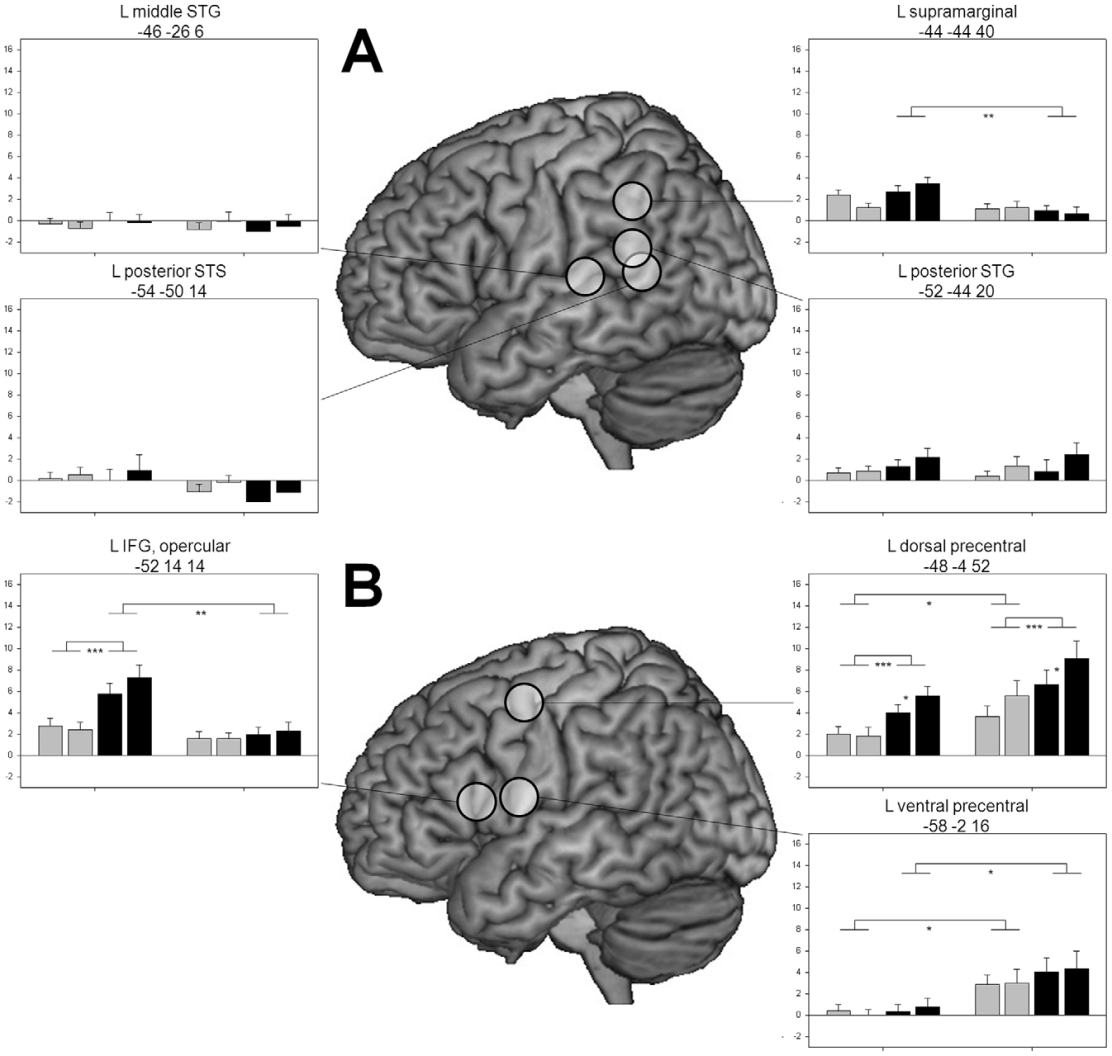
Activation in left hemisphere language regions. (A) Left temporo-parietal and (B) left frontal ROIs. Same captions as in Figure 2.

As noted in the Introduction, reviews of imaging studies assume that dysfunctions of posterior language areas are of primary importance for dyslexic reading problems. For selection of ROIs in the posterior superior temporal gyrus (STG) and superior temporal sulcus (STS), we relied on coordinates from our meta-analysis [41] and added a middle STG region which recently was found to exhibit underactivation in adult dyslexics in response to a task requiring the integration of letters and speech sounds [42]. Furthermore, a region in the supramarginal gyrus (SMG) was based on the finding of dyslexic underactivation in response to pseudowords in the whole-brain analyses. Figure 3A shows the location of these ROIs and the extracted signal change estimates. Several findings are remarkable: First, there was no reliable activation compared to baseline in the middle STG and the posterior STS, and activation levels were also low in the posterior STG with only long pseudowords differing from baseline. In the SMG, corresponding to the whole-brain analysis, dyslexic readers exhibited reduced activation in response to pseudowords, *p*<.01.

#### Left frontal regions

The whole-brain analysis in response to pseudowords revealed dyslexic underactivation in triangular and opercular parts of the IFG and dyslexic overactivation in precentral regions and the SMA. The ROIs were based on peaks of these clusters (Figure 3B). For the ROI in the IFG, dyslexic readers failed to exhibit the marked increase of activation from words to pseudowords of the nonimpaired readers. In the ventral precentral ROI, dyslexic readers exhibited higher activation compared to controls for both words and pseudowords, *p*s<.05. A similar pattern was evident in the dorsal precentral ROI with again higher dyslexic activation for words, *p*<.05, and a tendency for pseudowords, *p* = .06. In addition, both groups here exhibited length effects on activation for pseudowords, *p*s<.05.

## Discussion

### Behavioral manifestation

The dyslexic participants of the present study exhibited the behavioral manifestation of dyslexia in regular orthographies, that is, they suffered from a severe impairment of reading speed but not of reading accuracy. As evident from Table 3, on the time-limited sentence reading test used for selection, they processed only about half as many sentences as nonimpaired readers. However, they hardly committed any errors. Similarly, for the accompanying reading aloud tests presenting lists of words and pseudowords, their reading rate was about half the rate of the controls, but accuracy even for pseudowords was close to ceiling with about 95% correct. Of specific importance for the behavioral similarity with acquired cases of letter-by-letter (LBL) readers is the abnormal word-length effect shown by the present dyslexic readers in the phonological lexical decision task (i.e., “Does xxx sound like a real word?”). Their response latencies increased by about 170 ms from short words (3–5 letters) to long words (6–10 letters). This latency increase was about three times the latency increase of the nonimpaired readers. This abnormal length effect speaks for a dysfunction of the lexical reading route (i.e., whole-word recognition and direct access to whole-word phonology) and reliance on serial sublexical orthographic-phonological coding instead. However, the abnormal length effect was not limited to words but was also observed for pseudowords. Here it may be due to reduced availability of multi-letter recognition units. Of importance is that the abnormal length effect on phonological lexical decisions in the scanner corresponds to the abnormal dyslexic length effect observed in reading aloud tasks and eye-movement studies mentioned in the Introduction.

**Table 3:** Means and standard deviations of participant characteristics.

	Nonimpaired readers	Dyslexic readers	
	(*n* = 18)	(*n* = 15)	*t* (31)
Age (years)	17.89 (1.13)	18.09 (1.12)	−0.51
Sentence reading			
Accuracy (% correct)	98.77 (0.76)	96.12 (3.91)	-
Speed (sentences correct/3 min)	53.17 (7.81)	26.17 (6.97)	10.38***
corresponding reading quotient	102.25 (11.08)	63.96 (9.89)	
Word reading			
Accuracy (% correct)	99.89 (0.32)	97.53 (2.20)	-
Speed (items/min)	123.28 (12.42)	72.80 (22.64)	7.72***
Nonword reading			
Accuracy (% correct)	99.06 (1.41)	95.09 (6.03)	-
Speed (items/min)	82.00 (13.24)	43.73 (16.13)	7.49***
Verbal IQ			
Vocabulary	118.33 (9.70)	103.67 (10.08)	4.25***
Similarities	114.44 (8.73)	106.33 (10.26)	2.46*
Digit Span	101.39 (11.48)	91.33 (10.26)	2.63*
Performance IQ			
Block Design	110.00 (7.48)	112.67 (12.37)	−0.76
Visual Puzzles	109.17 (14.48)	110.67 (15.22)	−0.29
Coding	105.28 (11.31)	97.33 (14.00)	1.80

Statistically reliable group differences are indicated by asterisks.

*p<.05,

***p<.001.

### Evidence for a left OT dysfunction

In the Introduction we hypothesized that the slow serial reading of our developmental dyslexia cases similar to the serial reading of acquired LBL readers may be due to a dysfunction of the left occipito-temporal (OT) cortex and specifically of the Visual Word Form Area (VWFA). Specific expectations were based on findings with nonimpaired readers showing that the left OT cortex is engaged by lexical route processes (i.e., storage of orthographic word representations and use of such representations for whole-word recognition), but even more so by sublexical route processes [39], [43]. Accordingly, we expected a left OT dysfunction to become evident as a failure of dyslexic readers to exhibit the “normal” length effect for pseudowords on OT activation. A dysfunction of left OT regions for visual word processing should also be reflected in generally reduced activation of left OT regions compared to activation shown by nonimpaired readers.

Both expectations found support. Specifically, dyslexic readers did not show the pseudoword-length effect of the nonimpaired readers in left OT sulcus regions which correspond to the VWFA. Absence of this length effect is remarkable as the effect of pseudoword-length on response latencies was larger for dyslexic than for nonimpaired readers. Furthermore, absence of a pseudoword-length effect in left OT sulcus regions stood in contrast to presence of this effect in a posterior fusiform region. It is also of interest that there was no word-length effect on left OT activation of dyslexic readers. Such an effect could have been expected as dyslexic readers – different from nonimpaired readers – exhibited a strong word-length effect on response latencies. The present finding that dyslexic readers failed to exhibit modulation of left OT activation in response to the length of words and pseudowords corresponds to previous findings showing that dyslexic readers failed to exhibit the increased left OT activation of nonimpaired readers to sublexical processing required by pseudohomophones, pseudowords, or low-frequency words [37], [38], [44], [45].

Dyslexic readers not only failed to exhibit length effects on activation in left OT regions, they also showed reduced activation of this region compared to nonimpaired readers. For words, the whole-brain analysis identified a small region with underactivation centered at (MNI-coordinates) x = −42, y = −46, z = −16. For pseudowords, a similarly centered but much larger region was identified (x = −44, y = −48, z = −18). In response to pseudowords, underactivation of the dyslexic readers was not limited to left OT sulcus regions, but was also found in posterior aspects of the inferior temporal gyrus and of the middle temporal gyrus. One may note that the reduced left OT activation of the dyslexic readers in response to words and to pseudowords was still substantial compared to fixation baseline. A more demanding visual baseline may have further reduced left OT activation shown by dyslexic readers in response to visual words. This possibility is suggested by the results of two recent studies which used complex visual displays as baseline and found dyslexic readers to exhibit no reliable left OT activation in response to visual word processing [37], [45]. The present findings add to the still limited evidence for a specific dysfunction of left OT regions in dyslexic readers. Although the quantitative meta-analysis by Richlan et al. [41] did find a local maximum of underactivation corresponding to the VWFA, only three out of 17 studies reported foci of underactivation in regions corresponding to the VWFA and altogether only six studies reported underactivations in a larger OT reading system including posterior inferior and middle temporal regions in addition to ventral OT regions.

From a similarity of developmental dyslexia and acquired LBL reading one would not only expect a functional abnormality of left OT regions but also an anatomical abnormality. Several recent studies, including our own, found reduced gray matter volume in the left OT cortex in dyslexic readers [46]–[48]. Interestingly, Frye et al. [46] divided the observed abnormalities in gray matter volume into reductions of cortical surface area and reductions of cortical thickness. It is assumed that cortical surface area is determined prenatally, whereas cortical thickness is determined during postnatal development. For the left OT cortex, dyslexic brain abnormalities were only found in terms of reduced cortical surface area and not in terms of cortical thickness. This suggests that the dyslexic brain abnormalities in the left OT cortex arise early during brain development and are not a consequence of the reduced amount of reading experience in dyslexics. Apart from the OT cortex, other gray matter abnormalities have been found in the cerebellum, the right superior temporal gyrus, and an anterior portion of the left inferior temporal gyrus (e.g., [47]–[50]).

### Occipital underactivation

An unexpected finding was functional abnormalities in occipital regions. Specifically, in response to both words and pseudowords dyslexic readers exhibited underactivation in the right inferior occipital gyrus, and in response to pseudowords there was additional underactivation in medial occipital regions (lingual gyrus and cuneus). These occipital underactivations are unexpected given the much prolonged processing time of the dyslexic readers. They also differ from previous findings of overactivation in occipital regions [35], [38]. One may reason that the long letter strings (6–10 letters) among the present items led to a left-to-right visual scanning strategy among the dyslexic readers. Such a focus on word-initial letters reduces information in the left visual field which projects to right occipital cortex. Reliance on left-to-right letter string scanning can be expected to have a marked length effect on regions engaged by serial grapheme-phoneme coding and phonological assembly. In this perspective, the failure of dyslexic readers to exhibit a pseudoword-length effect on left OT activation is remarkable. An abnormally strong length effect on activation was only identified in a left dorsal precentral region presumably engaged by silent articulatory processes.

### No evidence for a left TP dysfunction

As noted in the Introduction, in reviews of imaging research, the left temporo-parietal (TP) cortex is considered to be the prime locus of developmental reading difficulties by affecting self-reliant phonological word decoding based on serial grapheme-phoneme conversion [18]-[22]. Indeed, our quantitative meta-analysis of imaging studies [41] identified maxima of underactivation in posterior aspects of the superior temporal gyrus (STG) and the superior temporal sulcus (STS) and also in the inferior parietal lobule which, in some accounts, is subsumed under the left TP reading circuit [18], [21]. The present findings raise doubts on these assumptions. In response to pseudowords, nonimpaired readers exhibited no activation compared to fixation baseline in the left middle STG and left posterior STS, and, consequently, no underactivation of dyslexic readers could be observed. In the posterior STG, nonimpaired readers exhibited small but reliable activation compared to baseline, but so did dyslexic readers. Underactivation of dyslexic readers was only found in the left supramarginal gyrus (SMG). However, the whole-brain analyses raised doubts whether the SMG should be subsumed under the TP reading circuit. These analyses showed that the SMG activation of the nonimpaired readers was an extension of the high activation in a large intraparietal region which was quite distant from the sylvian fissure with absent or little activation.

### Left inferior frontal underactivation accompanied by precentral overactivation

Different from the absence of dyslexic underactivation in left posterior temporal regions, there was evidence for functional abnormalities in frontal language regions. The whole-brain analysis identified extended regions with marked underactivation in response to pseudowords (but not to words) in bilateral inferior frontal opercular regions and in a left inferior frontal triangular region. The ROI analyses showed that the underactivation in response to pseudowords was mainly due to a failure to exhibit the increase of activation from words to pseudowords which was shown by the nonimpaired readers. From the whole-brain analysis it is evident that the reduced responsiveness of IFG regions to pseudowords stood in marked contrast to overactivation in adjacent precentral regions and also to overactivation in the SMA and in a right middle frontal region. The overactivation in left premotor regions was accompanied by overactivation in several subcortical regions (e.g., putamen, caudate, thalamus) and in the cerebellum. Overactivation in subcortical regions was also a dominant finding in our previous studies with German dyslexic readers [35], [38] and may reflect the slow effortful reading of our dyslexic participants.

### A disconnection between left OT and left IFG?

A comparison of the ROI-based activation patterns in Figure 2 and Figure 3 shows an impressive similarity between left OT and left IFG regions as in both areas dyslexic readers failed to exhibit the increase of activation from words to pseudowords which was shown by the nonimpaired readers. Furthermore, they failed to exhibit the increase of activation from short to long pseudowords of the nonimpaired readers. These patterns suggest that in nonimpaired, but not in dyslexic readers, both the left OT and the left IFG regions were responsive to the increased demands of sublexical pseudoword reading. Recent studies of functional and effective brain connectivity in nonimpaired readers suggest that the left OT cortex is substantially involved in driving brain activation in left inferior frontal areas (e.g., [51]–[54]). There is also a first study which points to abnormalities in effective connectivity of left OT to left IFG regions in dyslexic readers [55]. Of main interest would be anatomical findings on integrity of white-matter tracts linking these regions. There are findings of white-matter abnormalities in dyslexic readers in the left hemisphere (e.g., [56]–[59]). However, it is yet under debate which fiber tracts are specifically affected (see [60]). A promising candidate fiber tract is the left superior longitudinal fasciculus linking OT and IFG regions. Future diffusion tensor imaging studies may shed light on this hypothesis.

### Conclusions

On the phonological lexical decision task used for measuring brain activation, the present German-speaking dyslexic participants, similar to acquired cases of letter-by-letter readers, exhibited an abnormal length effect on response times for both words and pseudowords. This abnormal length effect corresponds to their severely impaired reading speed. Corresponding to lesions of left occipito-temporal (OT) regions in acquired cases, we found a dysfunction of this region in our developmental cases who failed to exhibit any responsiveness of left OT regions to the length of words and pseudowords. This absent responsiveness in the left OT cortex was accompanied by absent responsiveness to increased sublexical reading demands in phonological inferior frontal gyrus (IFG) regions. In contrast, corresponding to slow effortful dyslexic reading, our dyslexic readers exhibited abnormally high engagement of left premotor, subcortical, and cerebellar regions. Interestingly, neither nonimpaired nor dyslexic readers showed activation in left superior temporal regions which – corresponding to the phonological deficit explanation – are considered the prime locus of reading difficulties. The present functional imaging results suggest that developmental dyslexia similar to acquired letter-by-letter reading is due to a primary dysfunction of left OT regions.

## Materials and Methods

### Participants

Fifteen German-speaking dyslexic adolescents and young adults (age range: 16–20 years) were added to the sample of nonimpaired readers of the Schurz et al. [39] study. All participants were male, right-handed, and had normal or corrected-to-normal vision. The study was approved by the ethics committee of the University of Salzburg (“Ethikkommission der Universität Salzburg”). Participants gave written informed consent and were paid for their participation.

Group assignment was based on performance on a reading speed test which presents a list of sentences from which as many as possible have to be marked as correct (making sense) or incorrect within three minutes. The content of these sentences is simple as the main aim of the test is to allow a quick assessment of reading speed impairments. In studies assessing the validity of similar published tests for school-children, correlations between sentence reading scores and reading aloud performance on subtests of our Salzburger Lese- und Rechtschreibtest [61] ranged from .76 to .81.

Participants were assigned to the dyslexic group if their reading speed score (correctly scored sentences) was below percentile 10. All the nonimpaired readers had exhibited a reading speed score above percentile 15. Percentiles were based on a preliminary norm sample of about 300 adolescents and young adults. As evident from Table 3, the dyslexic readers processed only about half of the number of sentences processed by the nonimpaired readers. Their mean reading quotient (*M* = 100, *SD* = 15) based on the norm sample was below 70, whereas that of the nonimpaired readers was about average. The close to perfect accuracy of the dyslexic sample in evaluating the sentences rules out that their low test scores may reflect an accuracy problem. Slow reading speed in the absence of an accuracy problem is also evident from the additional reading measures in Table 3 which characterize reading aloud lists of words and pseudowords with increasing difficulty (time-limit: one minute). The combined reading aloud scores were highly associated with the sentence reading scores, Spearman's *r*(33) = .91. For nonimpaired and dyslexic readers separately, these correlations were *r*(18) = .58, *p*<.05, and *r*(15) = .80, *p*<.001, respectively. In summary, reading speed of our dyslexic readers was about half the speed of the nonimpaired readers. In contrast, reading accuracy of the dyslexic sample was close to perfect even for pseudowords.

A further inclusion criterion for the dyslexic group was a nonverbal IQ score in the normal range (i.e., at least 90). Nonverbal IQ was measured by three subtests (Block Design, Visual Puzzles, and Coding) of the German adaptation [62] of the Wechsler Adult Intelligence Scale-Revised (WAIS-R). In addition to the Performance Scale subtests, three subtests (Vocabulary, Similarities, and Digit Span) of the Verbal Scale were presented. The means in Table 3 show that dyslexic readers exhibited lower scores on all three verbal subtests but on none of the performance subtests. However, with exception of the Digit Span subtest, the mean scores of the dyslexic sample on the Vocabulary and the Similarities subtest were still above average.

### Stimuli and Task

Stimuli and task were identical to Schurz et al. [39] where an item list is provided. The task was to decide whether an item sounds like an existing word and the stimulus set consisted of 180 German words (mostly nouns), 180 pseudohomophones derived from these words, and 180 pronounceable pseudowords. Half of the items from each category were referred to as short (consisting of 3 to 5 letters) and the other half as long (consisting of 6 to 10 letters). Examples for short items of the three categories are “Text” (text) – “Tekst” – “Tokst”. Examples for long items are “Produktion” (production) – “Broduktion” – “Proklinom”. Actually, 69 out of 180 pseudohomophones were constructed by exchanging B/P, D/T and G/K in word-initial position but only when followed by L or R. In these consonant clusters, the aspiration of the unvoiced stops is largely lost and this is specifically so in the Southern variant of German. From our previous experience with the phonological lexical decision task [63], [64] we knew that participants sometimes find it hard to believe that a “misspelling” (pseudohomophone) sounds exactly like the intended word. Therefore, during a familiarization with the task outside the scanner, participants were instructed to accept a “misspelling” as sounding like the intended word even when they (wrongly) felt that the pronunciation may not be fully correct. A substantial number of examples for “misspellings” were presented. However, this familiarization was of only limited success. Specifically, short pseudohomophones (with reduced letter overlap with the correct spelling) led to substantial numbers of wrong NO responses. Accuracy for short and long pseudohomophones was 73% and 79% for dyslexic readers, and 79% and 89% for nonimpaired readers, respectively. Furthermore, the specific difficulty of the short pseudohomophones may have been responsible for the absence of a reliable length effect in both dyslexic (short: 1206 ms, long: 1270 ms) and nonimpaired readers (short: 1015 ms, long: 1010 ms). Since the main focus of Schurz et al. [39] was on the brain reflection of a length by lexicality (familiar vs. unfamiliar letter strings) interaction, pseudohomophones were deleted and this was also done in the present study. However, there is little reason to suspect that the difficulty of pseudohomophones does affect processing of pseudowords on which – together with words – the present analyses are based. Importantly, the phonological instruction (i.e., “Does xxx sound like a real word?”) and the presence of the pseudohomophones prevent that NO responses to pseudowords are simply based on orthographic (un)familiarity.

Each item was presented for 1260 ms with an inter-stimulus interval of 1360 ms during which a fixation cross was shown. YES responses (for words and pseudohomophones) were given by button press with the right index finger and NO responses (for pseudowords) with the right middle finger.

Table 4 shows item characteristics for short and long words and pseudowords. As evident from the means, the critical length manipulation was close to identical for words and pseudowords in terms of number of letters, number of syllables, and bigram frequency. Short and long words were matched for frequency of occurrence in written and spoken language and in number of orthographic neighbors (same-length words differing by one letter) based on the CELEX database [65]. However, long pseudowords had fewer orthographic neighbors than short ones (Mann-Whitney *U* test: z-value = 4.94, *p*<.001). In absolute terms, the difference was small (1 neighbor) and very small compared to orthographic neighborhood size differences used in studies which found a neighborhood size effect on brain activation (e.g., [66], [67]). Importantly, the smaller number of orthographic neighbors of long compared to short pseudowords should have made the correct NO response easier. This effect could only have been very small as long pseudowords led to less correct NO responses than short ones in both groups as the combined accuracy percentages were 86% and 81% for short and long pseudowords, respectively. Furthermore, as evident from Table 1, latencies of NO responses were substantially prolonged for long compared to short pseudowords.

**Table 4:** Means and standard deviations of item characteristics.

	Short words	Long words	Short pseudowords	Long pseudowords
Number of letters	4.5 (0.6)	7.5 (1.2)	4.5 (0.6)	7.5 (1.1)
Number of syllables	1.3 (0.5)	2.1 (0.6)	1.3 (0.5)	2.2 (0.6)
Frequency per million	1.2 (0.7)	1.2 (0.6)	-	-
Coltheart's Neighbors	1.3 (1.1)	1.3 (1.5)	1.3 (1.7)	0.4 (0.8)
Bigram frequency	18638 (18088)	79972 (37815)	15463 (15928)	66639 (38876)

A fast event-related design was used to investigate the hemodynamic response to the different types of stimuli. In order to avoid that a participant had to evaluate both, a base-word and its pseudohomophone, participants were presented one of two item sequences which were designed such that for each word-pseudohomophone pair, one sequence contained the base-word and the other its pseudohomophone. Each of these sequences included only 90 items per category (45 short and 45 long ones). Presentation of the items was divided into 3 runs (90 items each) with each run additionally containing 25 null-events with a fixation cross in the middle of the screen. The runs were separated by short breaks. The order of items and null-events within each run was determined by a genetic algorithm [68] which selects the most efficient sequence for testing stimulus contrasts. The stimulus onset asynchrony of 2620 ms is not a multiple of the TR of 2200 ms which enhances the efficiency of sampling the hemodynamic response at different time points. Before the experiment started, practice trials were used to familiarize participants with the task. Stimulus delivery and response registration was controlled by Presentation (Neurobehavioral Systems Inc., Albany, CA, USA).

### Image acquisition and analysis

Data were obtained with a Philips Gyroscan NT 1.5 Tesla scanner (Philips Medical Systems Inc., Maastricht, the Netherlands). Functional images sensitive to blood oxygen level dependent (BOLD) contrast were acquired with a T2* weighted gradient echo EPI sequence (TR 2200 ms, TE 45 ms, matrix 64×64 mm, FOV 220 mm, Flip Angle 90°). 25 Slices with a slice thickness of 5 mm and a slice gap of 0.7 mm were acquired within the TR. Scanning proceeded in 3 sessions with 146 scans per session. In addition, a high resolution (1×1×1.2 mm) structural scan was acquired from each participant with a T1 weighted MPRAGE sequence. For preprocessing and statistical data analysis, SPM5 software was used (http://www.fil.ion.ucl.ac.uk/spm) running in a MATLAB 6.5 environment (Mathworks Inc., Sherbon MA, USA). Functional images were realigned and unwarped, slice time corrected and then coregistered to the high resolution structural image. The structural image was normalized to the MNI T1 template image, and the resulting parameters were used for normalization of the functional images, which were resampled to isotropic 3×3×3 mm voxels and smoothed with a 6 mm FWHM Gaussian kernel.

Statistical analysis was performed in a two stage mixed effects model. In the subject-specific first level model, each stimulus onset was modeled by a canonical hemodynamic response function and its temporal derivative. Only correctly answered trials were included in the analysis. The incorrect answers and missed trials were modeled as covariates of no interest. The functional data in these first level models were high pass filtered with a cut-off of 128 seconds and corrected for autocorrelation by an AR(1) model [69]. In these first-level models the parameter estimates reflecting signal change for short words vs. fixation baseline (which consisted of the interstimulus interval and the null events), long words vs. fixation, short pseudowords vs. fixation, and long pseudowords vs. fixation were calculated in the context of a GLM [70]. These subject-specific contrast images were used for the second level random effects analysis. Within-group contrasts (words vs. fixation, pseudowords vs. fixation) were examined by *t*-tests thresholded at *p*<.05 (corrected for multiple comparisons using the family-wise error rate) at the voxel-level combined with a minimum cluster extent threshold of at least 20 voxels. To reduce the multiple comparisons problem, these regions were used as masks to search for group differences. The group contrasts were thresholded at *p*<.005 (uncorrected) at the voxel-level combined with the same cluster extent threshold of 20 voxels as the within-group contrasts.
